# Sub-Inhibitory Concentrations of Rifampicin Strongly Stimulated Biofilm Production in *S. aureus*

**DOI:** 10.2174/1874285801711010142

**Published:** 2017-06-30

**Authors:** Agostinho Alves Lima-e-Silva, Renato Geraldo Silva-Filho, Henry Marcel Zalona Fernandes, Carmen Soares Meirelles Saramago, Alice Slotfeldt Viana, Maria José Souza, Eduardo Matos Nogueira

**Affiliations:** 1 Department of Microbiology and Parasitology Rio de Janeiro, Biomedical Institute, Federal University of the State of Rio de Janeiro (UNIRIO), Rio de Janeiro, Brazil; 2 IPPMG, Federal University of Rio de Janeiro (UFRJ), Rio de Janeiro, Brazil; 3 Federal Hospital of the State Servers, Rio de Janeiro, Brazil; 4 Laboratory of Genomic, Biomedical Institute, Federal University of the State of Rio de Janeiro (UNIRIO), Rio de Janeiro, Brazil

**Keywords:** *Staphylococcus aureus*, Biofilm, Sub-MICs, Rifampicin, Minocycline, Congo red

## Abstract

**Background and Objectives:**

*Staphylococcus aureus* is an important pathogen and a frequent cause of infections associated with biofilm production in implantable medical devices. Biofilm production can be induced by sub-inhibitory concentrations (sub-MICs) of certain antibiotics, but few studies have researched this occurrence in *S. aureus*. In this study, we investigated the effect of sub-MICs of rifampicin and minocycline on biofilm production by five clinical and five non-clinical *S. aureus* isolates.

**Methods:**

Microtiter Plate assay and Congo Red Agar Test were used to analyze the biofilm production. The biofilm composition was evaluated by the detachment assay with sodium metaperiodate and proteinase K.

**Results:**

Rifampicin sub-MICs induced very high biofilm formation in seven isolates that were non-producers in Tryptic Soy Broth. In one producer isolate, the biofilm formation level was not affected by sub-MICs of this drug. Sub-MICs of minocycline did not induce biofilm production in all isolates tested and in two producer isolates, instead, MIC/2 and MIC/4 inhibited biofilm production. The results of the drugs in combination were similar to those with rifampicin alone. The biofilm matrix was identified as polysaccharide, except for one producer isolate, classified as proteinaceous. Polysaccharide biofilm producer isolates, when grown on Congo Red Agar without sucrose, but with sub-MICs of rifampicin, showed results in agreement with those obtained in Microtiter Plate Test.

**Conclusion:**

The high biofilm production induced by sub-MICs of rifampicin has potential clinical relevance, because this is one of the drugs commonly used in the impregnation of catheters. In addition, it is used adjunctively to treat certain *S. aureus* infections.

## 1 INTRODUCTION

*Staphylococcus aureus* is an important community and nosocomial pathogen. The virulence of this microorganism is multifactorial and includes the ability to produce biofilm on tissues and medical devices. Biofilms are complex multicellular communities in which cells are encased in a polymeric matrix that confers additional drug resistance and protection against mechanisms of immune defense [1]. In staphylococci, biofilm formation is mediated by *icaADBC*-dependent and -independent pathways [2].

The genes of *ica* operon encode enzymes that are involved in the production, externalization and elongation of the polysaccharide intercellular adhesin (PIA), which mediates the intercellular adherence of bacteria and its accumulation in multilayer biofilms. Specific proteins can substitute PIA in cell–cell adhesion in the PIA-independent biofilm and, sometimes, extracellular DNA (eDNA) can be the main component of the matrix [1, 3]. Biofilm formation occurs after the initial binging of cells to biotic or abiotic surfaces coated with plasma proteins, such as fibronectin or fibrinogen [4].

Because of the large number of patients suffering from biofilm-based device-related infections, several strategies for their prevention have been developed, such as the use of vascular and urinary catheters impregnated with antimicrobials [5]. A common method of antimicrobial coated catheters involves the use of rifampicin and minocycline. This combination was effective in preventing the colonization of catheter surfaces by slime-producing *S. epidermidis* and *S. aureus* isolates and displayed broad-spectrum inhibitory activity against other microorganisms [6].

Minocycline seems to prevent the development of rifampicin resistant mutants [7], while rifampicin exhibits good activity against staphylococci in biofilm, being able to penetrate it and kill organisms in the sessile phase of growth. Monotherapy with rifampicin has been abandoned, because of the rapid development of resistance. However it has been used adjunctively to treat *S. aureus* infections [8].

Sub-inhibitory concentrations (sub-MICs) of certain antibiotics can influence the staphylococcal biofilm expression, with induction or increase of biofilm production [9]. During the use of antimicrobial coated catheters, this effect may be predictable and can compromise the goal of using them. Thus, this study was conducted to examine the effects of rifampicin and/or minocycline sub-MICs on biofilm formation by some clinical and non-clinical *S. aureus* isolates.

## 2 MATERIALS AND METHODS

### 2.1 Bacterial Isolates

Twenty clinical isolates of *S. aureus* were obtained from different infected patients admitted to a hospital in Rio de Janeiro (HSE/RJ) and 20 non-clinical isolates from nasal swabs of healthy volunteers. The isolates were submitted to Gram staining, catalase and tube coagulase tests, while the identification and susceptibility to antimicrobial agents were performed with MicroScan WalkAway-96 System (Dade Behring Inc.). In addition, the isolates were tested for susceptibility to minocycline by the agar diffusion method and the resistance to oxacillin confirmed by cefoxitin disk screen test [10]. Specie confirmation was made by PCR assay [11]. All strains were screened for biofilm production using a microtiter plate assay (MTP assay), as described below. After initial screening tests, 10 isolates sensitive to rifampicin and minocycline, 5 from healthy volunteers (H01, H03, H39, H43 and H56) and 5 clinical isolates (C10, C12, C39, C40 and C50) were selected for subsequent tests. Both groups consisted of 4 biofilm non-producer and 1 producer isolates. The biofilm-producer *S. aureus* ATCC 43300 and the biofilm-negative *S. epidermidis* ATCC 12228 were used as reference strains. The Human Research Ethics Committee from HFSE approved this study with reference number 000.417.

### 2.2 Effect of Sub-MICs of Antibiotics on Biofilm Formation

The minimum inhibitory concentrations (MICs) for rifampicin or minocycline and for both drugs combined were determined by broth microdilution method, according to the Clinical Laboratory Standards Institute recommendations [10]. The effect of sub-MICs of the drugs tested on biofilm production was evaluated in MTP assay [12], with some modifications. Briefly, 1/2 serial dilutions of antibiotic stock solutions were prepared in tryptic soy broth (TSB-HiMedia) (4xMIC to MIC/32) and added (1:100) to overnight TSB cultures of isolates and reference strains. Controls were prepared in TSB without drugs. After homogenization, 200 µL of the bacterial suspensions per well were seeded in 96-well flat-bottomed polystyrene microtiter plates (Nunclon; Nunc A/S). After incubation for 24 h at 35ºC, the cultures were removed and wells were washed three times with distilled water. The attached bacteria were fixed with 200 µL of methanol for 15 min and the plates were emptied, air-dried and stained for 15 min with 200 µL of 2% Hucker's crystal violet solution. After removal of the dye, the plates were washed under running distilled water and air-dried. The dye bound to the adherent cells was extracted with 200 µL of 95% ethanol for 30 min and the optical density (OD_[A = 595nm]_) of the biofilm extracts was measured. All experiments were done in triplicate and repeated at least three times, and wells containing uninoculated TSB (blank) were included. The cut-off OD value (OD_c_) used to differentiate biofilm-producer and non-producer isolates was defined as three standard deviations above the mean OD of the blank [13]. Isolates with OD≤OD_c_ were considered as non-producers. The result of the OD average of biofilm extract was used to classify the isolates as weak producer (OD_c_<OD≤2xOD_c_), moderate producer (2xOD_c_<OD≤4xOD_c_) and strong biofilm producer (4xOD_c_<OD) [14].

### 2.3 Modified Congo Red Agar Test

The original composition of Congo Red Agar (CRA) [15] was modified, having been the medium prepared without sucrose. This medium was also prepared with the addition of rifampicin in concentrations equivalent to MIC/2 and MIC/4 obtained in broth microdilution method. The plates were seeded with 10 µL of overnight TSB cultures by spot plate technique, incubated aerobically for 24 h at 35°C and left overnight at room temperature. With this modification, the reaction was considered positive if the spots had dry crystalline consistency (rough), and negative if they had smooth appearance.The tests were performed in triplicate and repeated three times.

### 2.4 Biofilm Detachment Assay

The chemical nature of the biofilm matrix produced in the presence of rifampicin was determined by degradation with 40 mM sodium metaperiodate (Vetec, Brazil) or 1 mg/mL proteinase K (Sigma, USA) solutions in 0.1 M PBS (pH7.0), in a test resembling MTP biofilm assay [16]. The isolates were grown overnight in the TSB at 35ºC, diluted 1:100 in the TSB without and with drug (MIC/2 and MIC/4). Then, microtiter plates were seeded with 200 µL per well of the bacterial suspensions and incubated at 35^0^C for 24 hours. Subsequently, the cultures were removed and the wells washed with purified water. Degrading agents and PBS (control), 200 μL per well, were put in triplicate wells and the plates incubated for 2 h at 35ºC. Following, the wells were washed twice with distilled water, and the next steps were developed as described in MTP biofilm assay. A reduction over 50% in OD average, when compared to the control, of wells treated with degrading agents, indicated the chemical nature of the biofilm.

### 2.5 Detection of *icaA* and *icaD* Genes

Bacterial suspensions were prepared in ultrapure water with colonies grown in Tryptone Soya Agar (TSA - HiMedia, Mumbai, India) and cells lysed by boiling the suspension for 5 min, followed by thermal shock and centrifugation at 12.000xg for 5 min [17].

Supernatants were used as DNA template in a multiplex Polymerase Chain Reaction (PCR) for *icaA* and *icaD* genes detection. PCR reactions were performed with Taq DNA Polymerase Master Mix Red (Ampliqon A/S, Denmark), according to the manufacturer’s directions, in a LifePro Thermal Cycler (Hangzhou Bioer Technology Co.). For the detection of *icaA* gene, the primers were as follows: *ica9*-TCGCACTCTTATTGATAGTCGCTACGAG and *ica10*-TGCGACAAGAACTACTGCTGCGTTAAT [18]. The primers for *icaD* were designed from published sequence of the *icaD* locus of *S. aureus* ATCC 25923 in GenBank (acession number CP009361.1) using PrimerBLAST (http://www.ncbi.nlm.nih.gov/tools/primer-blast/): *icaD1*-TGGTCAAGCCCAGACAGAGGG and *icaD2*-TCGCGAAAATGCCCATAGTTTCA. The amplified products were analyzed by agarose (1.5%) gel electrophoresis with GelRed™ and visualized using UV light. Their sizes were estimated by comparison with 100bp DNA ladder (Invitrogen - Life Technologies, Canada).

## 3 RESULTS

### 3.1 Properties of Isolates

After screening for antimicrobial susceptibility and biofilm production 5 clinical (C10, C12, C39, C40 and C50) and 5 non-clinical isolates (H01, H03, H39, H43 and H56) of *S. aureus* were chosen. H56 isolate was classified as strong biofilm producer, and C39 as a weak producer. The other isolates were classified as non-producers. Among the non-clinical isolates, H43 was beta-lactamase positive and H56 was resistant to erythromycin and beta-lactamase positive. H01 isolate showed resistance to benzyl penicillin and oxacillin, and it was positive in cefoxitin disk screen test, being phenotypically characterized as community-associated meticillin-resistant *S. aureus* (CA-MRSA). The clinical isolates were positive for beta-lactamase and susceptible to other drugs tested.

### 3.2 Effects of Sub-Inhibitory Concentrations of Rifampicin and Minocycline on Biofilm Formation

Sub-MICs of rifampicin induced high biofilm production in seven *S. aureus* strains (three non-clinical and four clinical), that were biofilm non-producers in TSB. Three non-clinical isolates (H03, H39 and H43) showed OD_595_ values, respectively, of 2.715, 2.296 and 2.733 in the presence of MIC/2, and 2.590, 2.602 and 0.587 in MIC/4, with control values ranging from 0.161 to 0.261 Fig. (**1**). H01 isolate remained as non-producer when growing with sub-MICs of the drug (data not shown), and H56 isolate, previously characterized as strong producer, showed increase of OD_595_ values from 1.486 in control without drug to approximately 2.800 in MIC/2 and MIC/4.

**Fig. (2):**
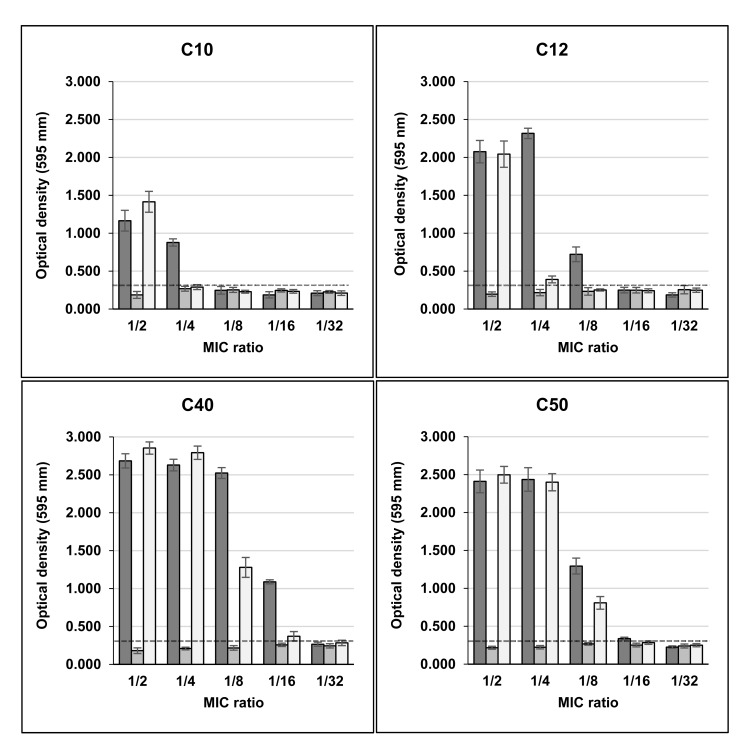
Biofilm production in microtitre plate assay by clinical isolates of S.aureus in TSB added of sub-inhibitory concentrations of rifampin, minocycline and rifampin+ minocycline. Dashed line indicates the cut-off OD (ODc) value. Average value of OD of control culture without drug: C10, C12, C40 and C50 < 0.300.

The same effect of high biofilm induction was observed in four clinical biofilm non-producer isolates (C10, C12, C40 and C50) in the presence of Sub-MICs of rifampicin. In MIC/2, the OD_595_ values for these isolates were, respectively, 1.165, 2.076, 2.684 and 2.411 and in MIC/4 0.878, 2.317, 2.629 and 2.436, with control values ranging from 0.205 to 0.243 (**Fig. 2**). C39 isolate maintained its weak biofilm production in the presence of sub-MICs of this drug (data not shown).

Unlike rifampicin, sub-MICs of minocycline did not induced biofilm production in clinical and non-clinical isolates that were previously classified as non-producers in TSB Figs. (**1** and **2**). Instead, MIC/2 and MIC/4 of minocycline inhibited biofilm production of H56 and C39 isolates, strong and weak biofilm producers, respectively.

The effect of rifampicin in combination with minocycline on biofilm production in clinical and non-clinical isolates was similar to that detected with rifampicin alone for almost all strains tested. However, the drugs in combination induced biofilm production in C10 isolate only in MIC/2, while for C12 isolate induction in MIC/4 was much lower than that of rifampicin alone (Fig. **2**).

### 3.3 Effect of Rifampicin Sub-MIC on Biofilm Formation in Modified CRA Test

**Fig. (3):**
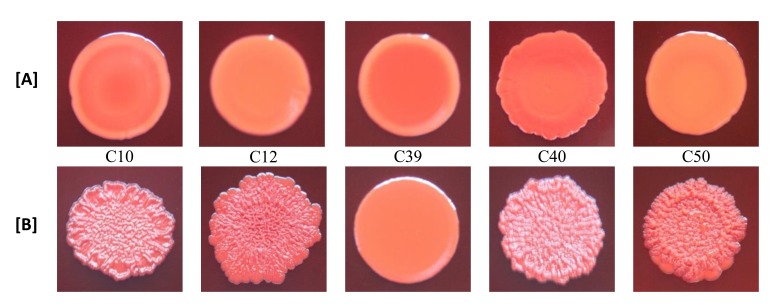
Clinical isolates of *S. aureus* growing in modified CRA (without sucrose) [A] and modified CRA containing sub-MICs of rifampicin (0.002 or 0.004 µg/mL) [B]. Identification of the isolates: C10, C12, C39, C40 and C50. Biofilm negative: spots with smooth appearance. Biofilm positive: spots with rough appearance.

**Fig. (4):**
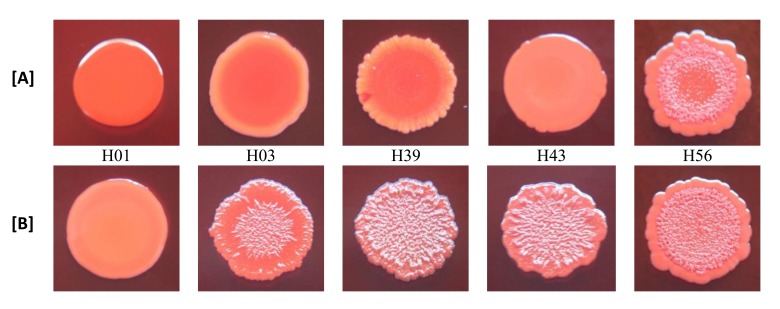
Non-clinical isolates of *S. aureus* growing in modified CRA (without sucrose) [A] and modified CRA containing sub-MICs of rifampicin (0.002 or 0.004 µg/mL) [B]. Identification of the isolates: H01, H03, H39, H43 and H56. Biofilm negative: spots with smooth appearance. Biofilm positive: spots with rough appearance.

With the exception of biofilm non-producer H01, which showed negative reaction in both media (modified CRA without and with rifampicin), all isolates classified as biofilm non-producers in MTP assay showed negative reaction in modified CRA and were positive in modified CRA with rifampicin. The strong producer H56 isolate showed positive reaction in both media, while the weak producer C39 isolate showed negative results (Figs. **3** and **4**).

### 3.4 Determination of Biofilm Chemical Nature

The chemical nature of the biofilm produced by the isolates was determined by MTP biofilm detachment assay. The biofilm producer strains H56 and C39 maintained their biofilm compositions in the presence of sub-MICs of rifampicin (polysaccharide and protein, respectively). The other strains, except for H1, produced polysaccharide biofilm in the presence of this drug. H1 isolate was not tested because it did not produce biofilm in medium with or without antibiotic.

### 3.5 Detection of *icaA* and *icaD* Genes

The presence of the *ica* locus was determined by multiplex PCR for the amplification of *icaA* and *icaB* genes, both essential for biofilm production in strains *ica*-dependent. These two genes were found in all strains studied.

## 4 DISCUSSION

Infections involving different types of implantable medical devices have been associated with bacteria embedded in biofilms, with *S. epidermidis* and *S. aureus* being among the most common etiologic agents of these infections [1, 8].

The staphylococcal biofilm expression is influenced by physical and chemical different factors, and it can be induced in response to external stress and sub-inhibitory concentrations of certain antibiotics [19-21]. Although this stimulation can be caused by low concentrations of antibiotics to sensitive or resistant bacteria [9, 22] we chose to investigate strains that were sensitive to the antimicrobials employed in the tests of biofilm production, *i.e.* rifampicin and minocycline.

The results of the present study demonstrated that sub-MICs of rifampicin induced high biofilm formation in both clinical and non-clinical *S. aureus* strains. Among the seven isolates classified as biofilm non-producers in TSB medium, six had very high induction rates in the presence of the drug, with increase in OD_595_ values ranging from 890% to 1580% in non-clinical isolates and from 420% to 1030% in clinical ones in MIC /2 or MIC /4. In two of these isolates (H39 and C43) the stimulus was so potent that continued until the value of MIC/16. Besides, in strong producer H56 isolate sub-MICs of rifampicin almost doubled biofilm production levels. However, the induction effect was not observed in all isolates.

The CA-MRSA strain maintained its phenotype of biofilm non-producer. Despite, in this isolate the *ica* operon was detected, as well as in all isolates, and it showed biofilm production when TSB was supplemented with 1.0% glucose (data not shown). Thus, this result indicates that this isolate has a stimulatory pathway of biofilm production that is not triggered by sub-MICs of rifampicin. The same explanation extends to C39 isolate, which did not change its weak producer phenotype in the presence of sub-MICs of rifampicin, but showed increased production (2x) in medium added with glucose (data not shown).

Few studies have investigated the effect of rifampicin sub-MICs in biofilm formation in staphylococci and they have shown diversified results. Most of the studies, unlike our findings, indicate inhibitory effects on biofilm formation. Schadow *et al*. [23] determined the adhesion properties of some strains of coagulase-negative staphylococci after treatment with sub-MICs of 14 antimicrobial agents. Compared with other agents investigated, rifampicin showed the greatest inhibitory effect in the adherence of *S. epidermidis* RP62A and RP12 strains. On the other hand, it caused an increase of 65% in adherence of strain RP14. Ozturk *et al*. [24] showed that sub-MICs of rifampicin determined decrease of biofilm production in most of the strong biofilm producer MRSA strains, and increase in a few strains. In turn, Mirani *et al*. [25] found no induction with rifampicin in biofilm formation in two MRSA isolates, one positive and one negative for *ica*A, on the contrary to what was observed for antibiotics active against cell wall.

In contrast to the results obtained for rifampicin, minocycline sub-MICs did not induce biofilm production in isolates classified as non-producers. Unlike, in H56 isolate, and to a lesser extent in C39, biofilm expression was inhibited. The comparison of our results with those of other studies is difficult, because these studies have focused primarily on the efficacy of this drug, alone or in combination, to eradicate *Staphylococcus* in preformed biofilms [26, 27].

However, considering the reports of low levels of other ribosome-targeting drugs on biofilm formation by *S. aureus*, but minocycline, the data have shown essentially inhibitory effects or no effect: roxithromycin [28]; linezolid, tetracycline, erythromycin [25]; azithromycin [29]. It is possible that the inhibition of biofilm production in *S. aureus* by sub-MICs of drugs with this mechanism of action be due to inhibition of the synthesis of proteins which are essential for the process of primary attachment in the first stage of biofilm formation, thus undermining the later stages.

Nevertheless, for coagulase-negative staphylococci, in addition to reports of inhibitory effect or no effect on biofilm formation by drugs which acts by inhibiting protein synthesis, except minocycline [22, 30], there are also studies showing the stimulation of this expression [9].

Analysing the results obtained with the combination of drugs in sub-MICs, we found that they were similar to those obtained with rifampicin alone. This suggests that the inducing stimulus for biofilm formation is mainly due to the action of rifampicin, and that this stimulus appears to supersede any eventual inhibitory effect of sub-MICs of minocycline in biofilm formation by the studied isolates.

The chemical nature of the biofilm matrix was characterized as polysaccharide in all isolates that showed induction or increase in biofilm production by sub-MICs of rifampicin, and these results are consistent with the *ica* operon detected in these strains. Moreover, considering that these isolates were identified as methicillin-sensitive *S. aureus*, the results are in agreement with those obtained by McCarthy *et al*. [2]. However, the weak producer C39 isolate, despite being positive for *ica* operon, showed no increase in the production of biofilm in the presence of rifampicin sub-MICs, and the chemical nature of its biofilm was proteinaceous in TSB with and without drug. Therefore, this isolate has a PIA-independent mechanism for the production of biofilm which is not affected by rifampicin, but that is stimulated by glucose as evidenced in additional experiment (data not shown).

In addition to the biofilms formed by PIA and eventually extracellular DNA, it is now recognized that several staphylococcal proteins can also promote the accumulation phase in an *ica*-independent manner [1]. Nevertheless, it is still not well established the type of matrix that can be produced in response to low concentrations of specific antimicrobials, and particularly if biofilm formation with protein matrix can be induced by sub-MICs of drugs such as rifampicin. A better knowledge of the chemical composition of the biofilms induced by different environmental conditions could be useful in the strategies for treatment of infections linked to the use of medical devices, since cells in biofilm with protein matrix have different organization of growing compared with those in PIA-dependent biofilm [31].

The results of the modified CRA test were in accordance with the biofilm production determined in MTP assay, except for weak producer isolate C39 that had negative results (smooth appearance) in modified CRA, with and without rifampicin, and were positive for biofilm production in MTP assay. This difference of results may be due to the low sensitivity of CRA test to detect weak biofilm producers [32]. Besides that, it should also be taken into account that the mechanism of positive reactions in CRA medium depends on polysaccharide biofilm production [15] and C39 isolate produced biofilm with protein composition.

In the original CRA assay [15], the results are based on black or red colour, and rough or smooth consistency of the cultures. In modified CRA test, such as formulated in our study, the criterion for a positive result was based solely on the appearance of rough spots, and not in the black colour. The purpose of this change was to test the isolate in the absence of substances capable of inducing the production of biofilm, except those in study. Because of this, sucrose, which could generate hyperosmolarity conditions that induce biofilm production, was not added. Thus, the conditions of the test were closer to what was executed in the MTP assay. Despite the limited number of isolates tested, our results indicate that CRA without sucrose may be useful to evaluate the potential of sub-MICs of antibiotics, such as rifampicin, to stimulate the production of polysaccharide biofilm in the absence of other inducing substances.

## CONCLUSION

In summary, our data reveal that, in contrast to minocycline, rifampicin sub-MICs were able to induce intense biofilm formation by isolates of *S. aureus*. Therefore, the adoption of procedures to prevent the exposure of bacteria to sub-inhibitory concentrations of this antimicrobial in clinical practice can contribute to reduce the occurrence of biofilm-related infections caused by this microorganism. Additionally, in the case of prolonged use of intravascular or urinary catheters impregnated with rifampicin, it should be considered that the possibility that sub-inhibitory levels of the drug could favour colonization by this pathogen if it gains access to these devices during their stay in the patient.

## Figures and Tables

**Fig. (1):**
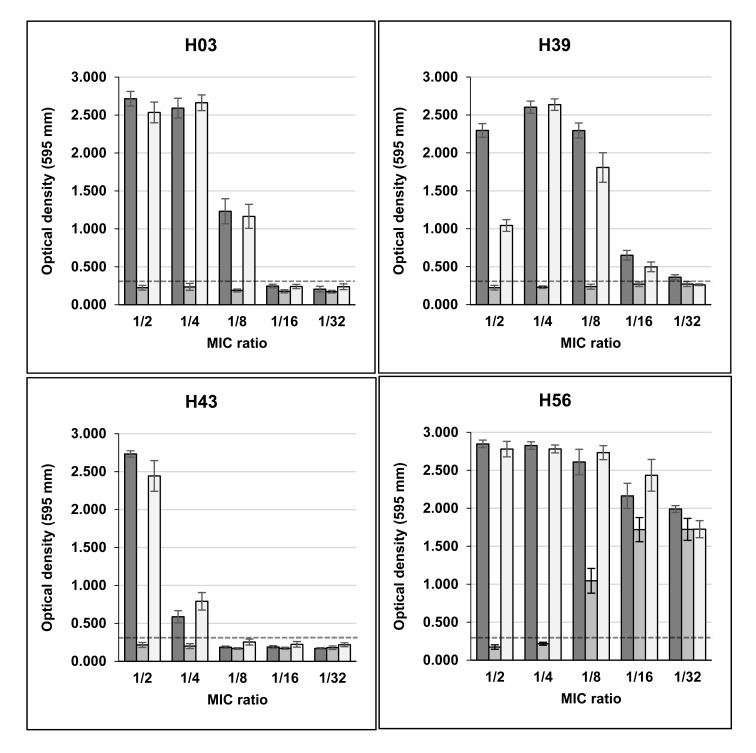
Biofilm production in microtitre plate assay by community carriage isolates of *S.aureus* in TSB added of sub-inhibitory concentrations of rifampin, minocycline and rifampin+ minocycline. Dashed line indicates the cut-off OD (ODc) value. Average value of OD of control culture without drug: H03, H39 and H43 < 0.300; H56=1.554.
